# Genomic and Phenotypic Landscape of Antibiotic Resistance in Gut Lactic Acid Bacteria from Livestock Environments

**DOI:** 10.3390/genes16121518

**Published:** 2025-12-18

**Authors:** Anna Mikołajczuk-Szczyrba, Karolina Wnęk-Auguścik, Paulina Średnicka, Dziyana Shymialevich, Ewelina Jaroszewska, Adrian Wojtczak, Agnieszka Zapaśnik, Joanna Bucka-Kolendo, Hanna Cieślak, Justyna Nasiłowska

**Affiliations:** 1 Department of Microbiology, Prof. Waclaw Dabrowski Institute of Agricultural and Food Biotechnology—State Research Institute, 36 Rakowiecka Street, 02-532 Warsaw, Poland; anna.mikolajczuk-szczyrba@ibprs.pl (A.M.-S.); diana.szymielewicz@ibprs.pl (D.S.); ewelina.jaroszewska@ibprs.pl (E.J.); adrian.wojtczak@ibprs.pl (A.W.); agnieszka.zapasnik@ibprs.pl (A.Z.); joanna.bucka@ibprs.pl (J.B.-K.); hanna.cieslak@ibprs.pl (H.C.); justyna.nasilowska@ibprs.pl (J.N.); 2 Department of Animal Environment Biology, Institute of Animal Sciences, Warsaw University of Life Sciences—SGGW, 8 Ciszewskiego Street, 02-786 Warsaw, Poland; karolina_wnek-auguscik@sggw.edu.pl

**Keywords:** antimicrobial resistance (AMR), antibiotic resistance genes (ARG), lactic acid bacteria (LAB), livestock microbiota, MIC testing, whole-genome sequencing (WGS), horizontal gene transfer

## Abstract

Background/Objectives: The widespread use of antibiotics in livestock has raised concerns about commensal gut bacteria, such as lactic acid bacteria (LAB), acting as reservoirs for antimicrobial resistance. This study aimed to characterize the antibiotic resistance profiles of LAB isolated from livestock feces by combining phenotypic susceptibility testing with whole-genome sequencing (WGS) to identify antibiotic resistance genes (ARGs) and their genomic context. Methods: Four LAB strains from farm animal fecal samples were subjected to antibiotic susceptibility testing for 9 antibiotics (ampicillin, gentamicin, kanamycin, clindamycin, chloramphenicol, erythromycin, streptomycin, tetracycline, and vancomycin) using MIC determinations. WGS was performed on each isolate to detect ARGs using curated databases and to determine the chromosomal or plasmid location of these genes. Results: All four isolates exhibited phenotypic resistance to at least one antibiotic class, most frequently to aminoglycosides. However, discrepancies between phenotype and genotype were noted: resistance to aminoglycosides was common despite the absence of known aminoglycoside-resistance genes, suggesting intrinsic, uptake-related mechanisms. In contrast, one strain carried the chromosomal *lsa*(D) gene but remained susceptible to clindamycin. WGS revealed that all strains harbored the chromosomal *van*(T) gene, while one isolate carried three additional plasmid-borne ARGs—*erm*(B), *cat*(A), and *tet*(W)—conferring resistance to macrolide–lincosamide–streptogramin antibiotics, chloramphenicol, and tetracycline. Another strain encoded *van(*Y), *lsa*(D), and *arr* on its chromosome. The detection of multiple plasmid-located ARGs in a single LAB isolate highlights their potential for horizontal gene transfer. Conclusions: This study provides a detailed phenotypic and genomic insight into antibiotic resistance in gut-derived LAB from livestock. The findings highlight that commensal LAB can harbor clinically relevant ARGs—sometimes on mobile genetic elements—without always expressing corresponding resistance phenotypes. Such LAB may serve as a hidden reservoir for antibiotic resistance, raising the risk of ARG dissemination through the food chain. These results underscore the importance of vigilant monitoring and genomic screening of LAB, especially those considered for use in foods or feed, to ensure they do not contribute to the spread of antimicrobial resistance.

## 1. Introduction

Antibiotics are antimicrobial compounds capable of inhibiting the growth of or eliminating bacteria and fungi. They are used in both human and veterinary medicine for the treatment and metaphylaxis of disease, to support weight gain, and to control zoonotic pathogens found in milk, eggs, meat, and products derived from them [1]. Antibiotics have served as growth promoters (AGPs) in animal production for more than seven decades in the United States, Australia, and multiple European countries [2]. According to reports, their use has been associated with a 4–8% increase in animal growth rate and a 2–5% improvement in feed conversion ratio (FCR), with some studies reporting improvements of up to 10% [3,4]. In response to the widespread—and often inappropriate—use of antimicrobials in food-producing animals, EU policy shifted toward evidence-based stewardship. EFSA conducts harmonized surveillance of antimicrobial resistance (AMR) across the food chain. The operational framework for monitoring in 2021–2027 is defined by Commission Implementing Decision (EU) 2020/1729 [5], which specifies target species/products, bacterial taxa under investigation, and sampling frequency. In addition, an annual joint EFSA–OECD report [6], framed within a One Health approach, synthesizes AMR trends in humans, food-producing animals, and meat. Despite a noticeable decline in antibiotic use in Europe, the global trend remains upward. In 2010, antibiotic consumption in livestock production was estimated at approximately 63,151 metric tons of active substances [7]. Current estimates place global antibiotic use in animal agriculture between 63,000 and 106,000 metric tons annually, with projections indicating an increase to 104,079 metric tons by 2030—an 11.5% rise compared to 2017 levels [8].

Although the use of antibiotics delivers tangible benefits in livestock farming, their responsible application—which encompasses the appropriate selection of agents, administration methods, and risk assessment—remains a significant challenge for many producers. Excessive antibiotic use, despite their pivotal role in disease control, fosters the development of AMR [9]. This phenomenon poses serious threats to both animal and human health, undermines the efficacy of treatments for infectious diseases, and heightens the risk of transmitting drug-resistant pathogens. AMR refers to the capacity of bacteria, fungi, viruses, and parasites to survive and proliferate despite exposure to therapeutic agents designed to eliminate them [10]. From an evolutionary perspective, bacteria adapt via chromosomal gene mutations or by acquiring exogenous resistance genes through horizontal gene transfer (HGT), which encode resistance factors [11,12]. The scale of infections caused by microorganisms resistant to antimicrobial agents has reached an alarming level in the 21st century and threatens global public health. Antibiotic resistance is widespread—it occurs in every country and affects all age groups. According to the opinion of the World Health Organization (WHO), AMR has emerged as one of the key threats to food security [13]. Worryingly, antibiotics and antibiotic resistance genes (ARGs) are prevalent in surface waters, treated wastewater, soil, animal waste, and even clinical settings, raising serious concerns among public health professionals [14,15]. Multidrug-resistant (MDR) bacteria—so-called “superbugs” resistant to multiple antibiotic classes—have been repeatedly detected in the environment and represent major threats to public health [16]. Moreover, the development and deployment of new antibiotics have significantly slowed since the 1980s, failing to keep pace with the rapid emergence of AMR. It is estimated that antibiotic-resistant infections may cause as many as 10 million deaths annually by 2050 [17].

Simultaneously, mounting evidence indicates continuous gene exchange between pathogenic strains and ostensibly harmless or even beneficial commensal bacteria. As a result, these commensals are increasingly regarded as reservoirs of ARGs, capable of disseminating them to pathogenic microorganisms and to other members of the gut microbiota [18,19]. However, many strains of lactic acid bacteria (LAB), particularly those from the genera *Lactobacillus*, *Bifidobacterium*, *Lactococcus,* etc., possess intrinsic or acquired resistance mechanisms that can protect them from antibiotics used therapeutically [19,20]. Exposure to antibiotics, even at low concentrations, can select strains harboring ARGs, thus increasing the risk of their persistence and dissemination within the gut microbiome. Therefore, it is essential to closely monitor the presence and characteristics of AMR in bacterial strains used in various applications, especially in the food industry and in medical applications, including dietary supplements, commercial microbial preparations, and fermented food production [21]. Research on the genetic stability of microorganisms intentionally used in the food chain, and their potential for transferring ARGs is now a key component of safety assessments [22]. Accordingly, the presence of AMR phenotypes in bacteria, the localization of ARGs, and their transferability have become focus areas for intensive investigation [23]. In light of the above, although LABs are widely used in the food chain—for example, in fermentation processes, food preservation, improvement of sensory properties, and various biotechnological and medical applications—their use requires caution, particularly with regard to their potential contribution to the dissemination of AMR [24,25].

The objective of this study was to characterise the AMR profiles of LAB isolated from the gut microbiota of livestock. To achieve this, isolated strains were identified and then subjected to phenotypic AMR testing and genotypic analysis of ARGs. Based on these data, the risks associated with the potential transmission of ARGs within the gut environment were evaluated, and implications for livestock health and food safety were assessed. Altogether, this work provides essential information for the safety assessment of LAB used in the food chain.

## 2. Materials and Methods

### 2.1. Biological Material

Fecal samples were collected from clinically healthy animals maintained under standard husbandry conditions and receiving routine veterinary care. The animals had not been treated with antibiotics or other antimicrobial agents in accordance with the legally required withdrawal periods. The biological material originated from a different production facility containing one flock or herd each of laying hens, broiler chickens, and beef cattle. To obtain representative samples, fresh feces were collected consecutively from four distinct locations within the bedding area at the same time. Samples were collected from two geographic locations, and detailed data are available in the MIGS (Minimum Information about a Genome Sequence) record. Sampling was performed using sterile instruments, which were replaced between sampling points to minimize the risk of environmental or cross-contamination. Immediately after collection, each sample was placed in a sterile, tightly sealed transport container and stored at 4 °C.

### 2.2. Isolation of Lactic Acid Strains

Fecal samples were first homogenized by manual mixing, after which 10 g of each sample was suspended in 90 mL of sterile phosphate-buffered saline (PBS) containing 0.05% Tween 80 (pH 7.2) (Merck, Darmstadt, Germany), preheated to 42 °C to facilitate dispersion. The suspensions were stirred manually for 2 min to ensure uniform homogenization prior to serial dilution and plating. The homogenized material was then serially diluted tenfold (10^−1^ to 10^−6^) in sterile PBS. From each dilution, 100 μL aliquots were spread, in duplicate, onto selective media commonly used for the isolation of LAB: MRS agar (Oxoid, Hampshire, UK) supplemented with 0.05% (*w*/*v*) L-cysteine (Pol-Aura, Dywity, Poland), TOS agar (Biomaxima, Lublin, Poland), and M17 agar (Merck, Darmstadt, Germany). The plates were incubated under anaerobic conditions at 37 °C for 48 h using an anaerobiosis jar with GasPak systems. After incubation, 71 colonies were selected based on characteristic morphological traits consistent with LAB, including small to medium size, circular shape, smooth and convex surface, entire margins, and white to cream coloration. The selected colonies were then subcultured onto fresh media of the same type to obtain pure cultures. The purified strains were preserved at −80 °C in cryotubes containing a 1:1 mixture of bacterial suspension and 25% glycerol for subsequent phenotypic and genotypic analyses.

### 2.3. MALDI-TOF MS Identification

To characterize the isolates, all previously selected colonies were subjected to preliminary identification using MALDI-TOF MS (Shimadzu AXIMA Confidence iD 5, Shimadzu, Kyoto, Japan). Material from 24 h fresh cultures was transferred onto a MALDI target well, followed by an on-spot protocol with 25% formic acid and α-cyano-4-hydroxycinnamic acid (α-CHCA) matrix according to the manufacturer’s instructions; each isolate was analyzed in three technical replicates. Quality control for each run included parallel analysis of the control strain *Escherichia coli* DH5α; a correct identification of the control was required for batch validity. Data processing and taxonomic assignment were carried out using SARAMIS Target (sample description, database version: 4.1.0.9) and SARAMIS Premium (analysis and result readout; bioMérieux, database version: 4.11), by matching acquired spectra against the reference library; genus/species-level identifications were accepted only when the manufacturer’s reliability thresholds were met.

### 2.4. DNA Isolation and 16S rRNA Identification

The LAB strains selected by the MALDI-TOF MS method were confirmed by sequencing of the *16S rRNA* gene region. Bacterial DNA was extracted using the commercial DNeasy PowerFood Microbial Kit (Qiagen GmbH, Hilden, Germany), as described by Kiousi et al. [26] and Bucka-Kolendo et al. [27]. Amplification was performed with the 16S-F (5′−AGAGTTTGATCCTGGCTCAG−3′) and 16S-R (5′−ACGGCTACCTTGTTACGACTT−3′) primers [28]. PCR conditions for gene amplification were as follows: an initial denaturation at 95 °C for 2 min, followed by 35 amplification cycles consisting of denaturation at 94 °C for 30 s, annealing at 51 °C for 35 s, and extension at 72 °C for 1 min. The process concluded with a final extension step at 72 °C for 10 min, performed using the SimpliAmp™ Thermal Cycler (Applied Biosystems™ ThermoFisher Scientific, Waltham, MA, USA). The purity of DNA was verified with a Nanopore ND-1000 Spectrophotometer (ThermoFisher Scientific, Watertown, MA, USA). The quality of the amplicons was checked by electrophoresis on a 2% agarose gel containing the SimplySafe™ interfering compound (5 µL/100 mL; EURx, Gdansk, Poland). To estimate the size of the amplicons, a DNA Ladder with a size range of 100–3000 bp (A&A Biotechnology, Gdansk, Poland) was used. Electrophoresis was conducted at 80 V for 30 min, in 1× TAE buffer, using the Sub-Cell GT Horizontal Electrophoresis System (Bio-Rad, Madrid, Spain). The bands were visualized using the GeneFlash Network Bio Imaging System (Syngene, Wales, UK). Following this, sequencing was outsourced to Genomed S.A. (Warsaw, Poland). The raw sequences were analyzed using BLASTn (NCBI 2.16.0) and deposited in the GenBank database.

The selected isolates were deposited in the Collection of Industrial Microorganisms—Microbiological Resources Center (KKP), Warsaw, Poland.

### 2.5. Phenotypic Antibiotic Susceptibility Profile

The phenotypic antibiotic susceptibility of LAB isolates was determined by measuring the minimum inhibitory concentration (MIC) for eight antibiotics: ampicillin, gentamicin, kanamycin, clindamycin, chloramphenicol, erythromycin, streptomycin, tetracycline, and vancomycin. The MIC values were determined for each isolate using E-test strips (bioMérieux, Marcy-l’Étoile, France) on MRS agar, according to the manufacturer’s instructions.

Individual colonies from the agar plates were picked and suspended in sterile saline to achieve a turbidity equivalent to a 0.5 McFarland standard. A sterile cotton swab was dipped into the cell suspension and used to inoculate the MRS agar plate by swabbing in three directions. After the surface had dried, an E-test strip was placed on the agar plate, which was then incubated anaerobically.

The MIC, defined as the point of intersection between the edge of the inhibition ellipse and the E-test strip, was recorded after 48 h of incubation at 37 °C. The assay was performed in three repetitions, and each repetition included duplicate measurements.

The isolates were classified as resistant if their MIC values exceeded the MIC breakpoints established by the EFSA [29,30].

### 2.6. Whole-Genome Sequencing and Bioinformatics Analysis

Whole-genome sequencing of the bacterial isolates was commissioned by GenXone SA (Złotniki, Poland). DNA libraries were prepared according to the manufacturer’s protocol using the Rapid Barcoding Kit reagents (Oxford Nanopore Technologies, Oxford, UK). Sequencing was performed on a GridION X5 platform (Oxford Nanopore Technologies, Oxford, UK) under the control of MinKnow v22.10.5. Basecalling and barcode demultiplexing were carried out using Guppy v6.3.8, generating individual FASTQ files for each barcode. De novo genome assemblies were obtained using Flye v2.9, followed by polishing with Medaka v1.7.2. Genome annotation was performed with Bakta v1.8.3, employing the latest RefSeq bacterial protein database. Genome completeness and contamination were evaluated with CheckM v1.2.2, and taxonomic classification was assigned using GTDB-Tk v2.3.2 with the GTDB release R220 (19 October 2024) database. Average nucleotide identity (ANI) values between isolates and reference genomes were computed with FastANI v1.3.3 to confirm species-level identity. ARGs and additional genetic determinants related to metal tolerance, stress response, and virulence were identified using AMRFinderPlus v3.12.15 (NCBI, Bethesda, MD, USA) and cross-validated against the Comprehensive Antibiotic Resistance Database (CARD, release 2024). Functional annotation was complemented with eggNOG-mapper v2.1.9, providing COG and KEGG orthology assignments for metabolic pathway reconstruction. Whole-genome maps and comparative visualizations were generated using Proksee v1 (https://proksee.ca, accessed on 6 November 2025).

## 3. Results

### 3.1. Identification

Among the 71 isolates analyzed by MALDI-TOF MS, 24 were identified as members of the family *Lactobacillaceae*, and 9 as belonging to the genus *Enterococcus*, including *Enterococcus hirae* (6 isolates), *Enterococcus faecium* (1 isolate), *Enterococcus faecalis* (1 isolate), and *Enterococcus durans* (1 isolate). In addition, isolates belonging to *Bacillus* spp. (2 isolates), *Escherichia coli* (4 isolates), *Proteus mirabilis* (2 isolates), and *Streptococcus* spp. (3 isolates) were identified, while 27 isolates could not be reliably identified. All isolates identified by MALDI-TOF MS as LAB were further subjected to phylogenetic analysis of the 16S rRNA. The obtained results showed similarity to reference sequences of *Limosilactobacillus reuteri*, *Lactococcus garvieae*/*petauri, Ligilactobacillus salivarius* and *Ligilactobacillus agilis.* Based on the phenotypic analysis of antibiotic resistance profiles, three strains from the genus *Lactobacillus* and one strain from the genus *Lactococcus* were selected for WGS. Further WGS analysis allowed for the precise taxonomic classification of the isolates as *L. reuteri*, *Limosilactobacillus oris*, *L. agilis* and *L. petauri*. In the subsequent sections of the manuscript, we will refer to the taxonomy obtained from the WGS analysis. The results of bacterial identification obtained using the three applied methods—MALDI-TOF MS, 16S rRNA gene sequencing, and WGS—are presented in Table 1.

### 3.2. Genome Characteristics

**Figure 1:**
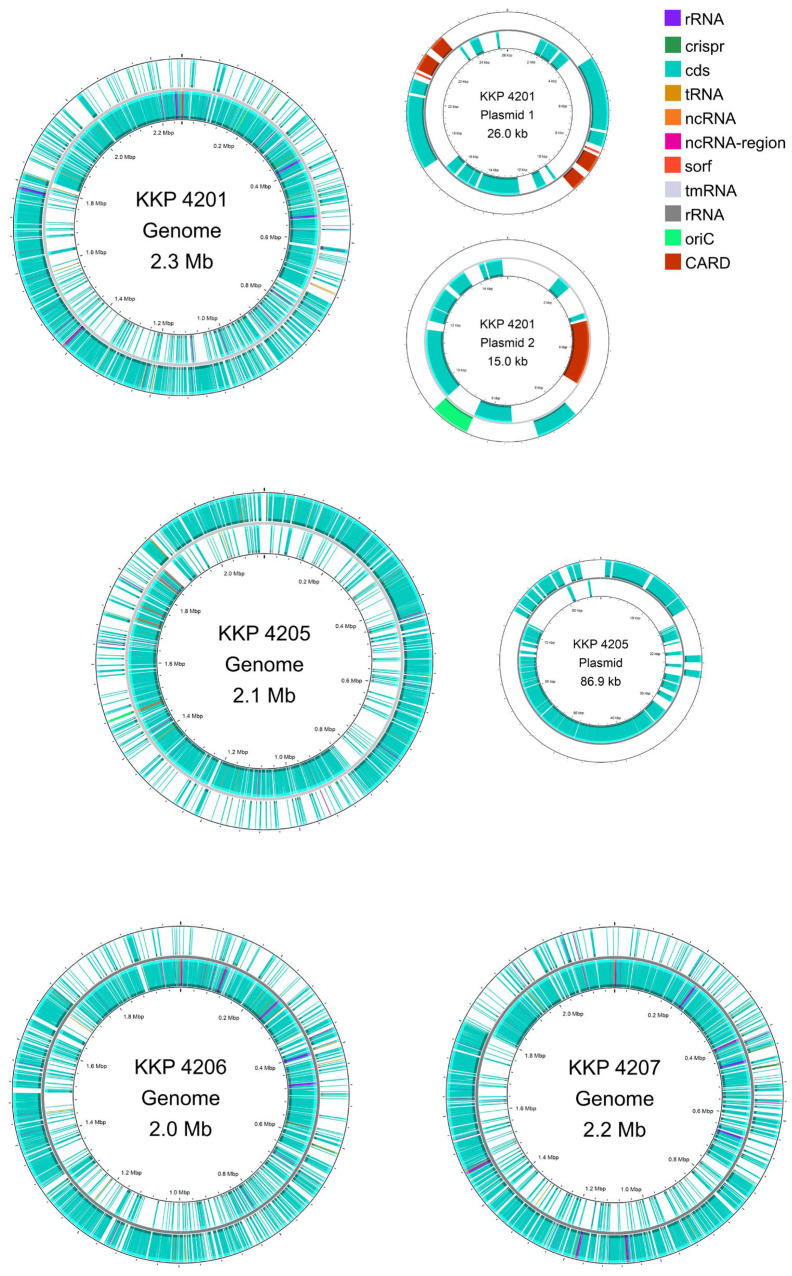
Genomic maps of the chromosome and plasmids of *L. reuteri* KKP 4201, *L. petauri* KKP 4205, *L. oris* KKP 4206, and *L. agilis* KKP 4207 constructed. Maps were generated using Proksee.

Strains *L. oris* KKP 4206 and *L. agilis* KKP 4207 each harbor a single circular chromosome (Table 2). The chromosome of *L. oris* KKP 4206 is 1,993,841 bp in length, whereas that of *L. agilis* KKP 4207 is 2,190,702 bp, with GC contents of 49.71% and 41.77%, respectively. The WGS of strain *L. petauri* KKP 4205 comprises a chromosome of 2,228,894 bp (GC content: 38.10%) and a single plasmid of 86,902 bp. Strain *L. reuteri* KKP 4201 carries a chromosome of 2,306,818 bp (GC content: 38.98%) and two plasmids, measuring 15,048 bp and 26,035 bp, respectively. Circular maps of the chromosomes and plasmids for all strains are shown in Figure 1.

### 3.3. Functional Annotation

The annotated genes of the four strains were classified into 20 COG categories (Table 3). The most abundant COGs in the majority of the strains were Translation (class J), Replication and Repair (class L), and Transcription (class K). Notably, a substantial proportion of genes in all four strains were also assigned to Carbohydrate Metabolism and Transport (class G), Cell Wall/Membrane/Envelope Biogenesis (class M), and Amino Acid Metabolism and Transport (class E).

### 3.4. Phenotypic Antibiotic Susceptibility Profile

The phenotypic antibiotic susceptibility testing of the examined LAB strains revealed diverse resistance profiles (Table 4). All tested strains, except *L. reuteri* KKP 4201, were susceptible to ampicillin, with MIC values ranging from 0.125 to 1 mg/L. *L. reuteri* KKP 4201 exhibited a high level of resistance to this antibiotic (MIC > 256 mg/L). Regarding aminoglycosides, a broad range of MIC values was observed. *L. petauri* KKP 4205, *L. reuteri* KKP 4201, and *L. agilis* KKP 4207 were resistant to gentamicin (MIC 24 ≤ 256 mg/L), whereas *L. oris* KKP 4206 remained susceptible (MIC 8 mg/L). All strains were resistant to kanamycin (MIC > 256 mg/L). For streptomycin, *L. petauri* KKP 4205, *L. reuteri* KKP 4201, and *L. agilis* KKP 4207 were resistant (MIC ≥ 1024 mg/L), while *L. oris* KKP 4206 remained susceptible (MIC 48 mg/L). Most isolates were susceptible to erythromycin (MIC ≤ 1 mg/L), except for *L. reuteri* KKP 4201, which exhibited high resistance (MIC > 256 mg/L). A similar pattern was observed for clindamycin: *L. petauri* KKP 4205, *L. oris* KKP 4206, and *L. agilis* KKP 4207 were susceptible (MIC 0.38–2 mg/L), whereas *L. reuteri* KKP 4201 was resistant (MIC > 256 mg/L). With respect to tetracycline, two out of four tested strains—*L. oris* KKP 4206 (MIC 16 mg/L) and *L. reuteri* KKP 4201 (MIC > 256 mg/L)—showed resistance, while the remaining two strains were susceptible (MIC 0.38–3 mg/L). For chloramphenicol, *L. reuteri* KKP 4201 (MIC 48 mg/L) was resistant, whereas *L. petauri* KKP 4205, *L. oris* KKP 4206, and *L. agilis* KKP 4207 were susceptible (MIC 0.5–3 mg/L). Antibiotic susceptibility testing for vancomycin was also performed for all strains, despite the lack of EFSA recommendations (Table 1) for some of them. In this case, almost all strains exhibited high resistance (MIC > 256 mg/L), except for *L. petauri* KKP 4205, which was susceptible (MIC 1.5 mg/L). In summary, the highest frequency of resistance was observed among aminoglycosides (gentamicin, kanamycin, and streptomycin), with resistance detected in all tested strains. In most cases, the isolates were resistant to all three aminoglycoside antibiotics, except for strain *L. oris* KKP 4206, where resistance was observed only to kanamycin. The tetracycline resistance phenotype was identified in two strains. Notably, strain *L. reuterii* KKP 4201 exhibited an MDR phenotype, demonstrating resistance to all antibiotics tested.

### 3.5. Genotypic Antibiotic Susceptibility Profile

In accordance with EFSA guidelines [22], ARGs were identified based on WGS using two curated databases: AMRFinder and CARD. Analysis of four strains (*L. reuteri* KKP 4201, *L. petauri* KKP 4205, *L. oris* KKP 4206 and *L. agilis* KKP 4207) revealed the presence of genes conferring resistance to various classes of antibiotics (Table 5).

In the genome of *L. reuteri* KKP 4201, four resistance genes were detected: *van*(T) (*vanG* cluster), *erm*(B), *cat*(A), and *tet*(W). The chromosomally encoded *van*(T) confers resistance to glycopeptides, while *erm*(B) (resistance to macrolides, lincosamides, and streptogramins) and *cat*(A) (resistance to chloramphenicol) were located on plasmid 1, whereas *tet*(W), associated with tetracycline resistance, was identified on plasmid 2. Strain KKP *L. petauri* 4205 harbored four chromosomal resistance genes: *van*(T) (*vanG* cluster) and *van*(Y) (*vanB* cluster), both conferring resistance to glycopeptides, *lsa*(D), which mediates resistance to lincosamides, streptogramins, and pleuromutilins, and *arr*, associated with rifamycin resistance. No resistance genes were detected on the plasmid of this strain. In *L. oris* KKP 4206, a single chromosomal gene, *van*(T) (*vanG* cluster), was present, conferring glycopeptide resistance. *L. agilis* KKP 4207 carried two chromosomal resistance determinants: *van*(T) (*vanG* cluster) and *qac*(G). While *van*(T) confers resistance to glycopeptides, *qac*(G) mediates resistance to quaternary ammonium compounds (QACs), such as benzalkonium chloride. In summary, the most frequently detected gene was *van*(T), present in all four strains and associated with glycopeptide resistance. Strain *L. reuteri* KKP 4201 exhibited the highest number of ARDs, with a total of four, including three plasmid-encoded genes (*erm*(B), *cat*(A*)*, *tet*(W*)*), suggesting a potential for horizontal transfer of these resistance determinants.

## 4. Discussion

ARGs present in livestock may enter the soil environment through the application of animal-derived fertilizers, potentially increasing the abundance of antibiotic resistance genes among soil microorganisms and posing a risk to animal and human health [31,32]. The occurrence of AMR in the ecosystem represents a complex ecological network [9,11] and carries the potential for HGT [10,12].

Excessive and often uncontrolled use of antibiotics in animal husbandry represents a major public health concern, directly contributing to the rise of AMR [1]. In livestock production, antibiotics are applied not only for therapeutic purposes but also metaphylactically, which favors the selection of resistant strains [2,3]. Of particular concern is the possibility that resistance acquired by commensal gut bacteria of farm animals may subsequently be transferred to other microorganisms through mobile genetic elements such as plasmids [9,10]. Animal feces, widely released into the environment, thus become a reservoir of ARGs and a potential source of their transfer to clinically relevant pathogens [5,9].

In this study, we focused on the isolation of LAB originating from fecal samples of farm animals. The antibiotic resistance profiles of four selected LAB strains were evaluated both phenotypically—by determining the MICs for the most commonly used antibiotics—and genotypically, using WGS. This integrative approach enabled us to determine whether the phenotypically observed resistance was supported by genomic evidence and to establish whether the resistance genes were located on the chromosome or on mobile genetic elements.

Our study revealed discrepancies between the phenotypic and genotypic profiles of AMR in the analyzed LAB strains (Table 6). In many cases, strains exhibiting phenotypic resistance lacked the corresponding ARGs, in agreement with previous reports [33,34]. All tested strains were resistant to at least one aminoglycoside, while three strains—*L. reuteri* KKP 4201, *L. petauri* KKP 4205, and *L. agilis* KKP 4207—exhibited phenotypic resistance to all three tested antibiotics in this class: kanamycin, gentamicin, and streptomycin, although in the case of gentamicin the resistance phenotype of strains *L. reuteri* KKP 4201 and *L. oris* KKP 4206 was low, yet still above the cutoff values established by EFSA [22]. However, WGS did not reveal the presence of any known aminoglycoside resistance genes in these strains. Similar findings were reported by Dec et al. [35], who analyzed 57 *Lactobacillaceae* isolates from pigeon feces and found that 89% were phenotypically resistant to kanamycin and 63% to streptomycin, whereas resistance genes were detected in only a few strains. Campedelli et al. [36] examined 182 reference *Lactobacillaceae* strains and observed phenotypic resistance to kanamycin in 61% of cases, while *aac*(3) and *aph*(3*)* genes, which may confer resistance, were found in only 11% of strains. In another study, Anisimova et al. [37] analyzed 20 lactic acid fermentation strains and reported resistance to kanamycin (100%), gentamicin (90%), and streptomycin (85%), yet genes encoding enzymatic modification of aminoglycosides, such as *aad*(A) and *aad*(E), were detected in only 20% of strains, and other known aminoglycoside resistance genes (*aac*(6′)-*aph*(2″), *aph*(3′)-III, *ant*(6), *ant*(2″)-I) were absent. Although rare, several *Lactobacillaceae* strains, including *L. plantarum*, *L. buchneri*, *L. agilis*, and *L. diolivorans*, have been reported to carry genes encoding enzymatic modification of aminoglycosides, such as *aac*(6′)-*aph*(2″), *ant*(6), or *aph*(3′)-III [35,36,38,39,40]. Notably, Stefańska et al. [39] detected the *aph(*3″)-IIIa gene in three *L. fermentum* strains with a MIC of 64 μg/mL, which were classified as kanamycin-sensitive. Aminoglycoside resistance in LAB is widely regarded as intrinsic and primarily associated with limited uptake of these antibiotics, which results from both the low permeability of the cell envelope and a reduced or absent transmembrane potential. This physiological feature is consistent with the lack of cytochrome-mediated electron transport systems in LAB, which in other bacteria facilitate the energy-dependent internalization of aminoglycosides. The importance of membrane impermeability in this intrinsic resistance has been further supported by observations showing that LAB cultured in bile-containing media exhibit increased susceptibility to aminoglycosides, indicating that enhanced membrane permeability facilitates antibiotic entry [37,39,41,42]. In Gram-positive bacteria, chromosomal mutations affecting membrane potential have also been documented, and LAB are known to display a high frequency of spontaneous mutations conferring resistance to kanamycin and streptomycin, underscoring the multifactorial and largely physiological nature of aminoglycoside resistance in this group of microorganisms [39].

Regarding β-lactam antibiotics (ampicillin), phenotypic resistance was observed only in the *L. reuteri* KKP 4201 strain, aligning with previous reports indicating that the majority of *Lactobacillaceae* are sensitive to β-lactams [37]. Despite the phenotypic resistance, no known β-lactam resistance genes were detected in this strain. Similar findings were reported by Dec et al. [33], who noted that 26% of the analyzed LAB isolates exhibited phenotypic resistance to ampicillin, yet the underlying genetic determinants could not be detected, leaving the mechanism responsible for this phenotype unresolved. Other studies likewise report the absence of the *bla* genes in LAB despite their phenotypic resistance to β-lactam antibiotics [33]. These observations suggest that LAB may possess alternative or as-yet-undescribed β-lactam resistance mechanisms or genes that have not been identified to date. Overall, the results highlight the need for further research aimed at elucidating potential novel resistance mechanisms in LAB strains and to better understand the discrepancies between phenotypic and genotypic β-lactam resistance.

Erythromycin resistance in our study was observed both phenotypically and genotypically exclusively in strain *L. reuteri* KKP 4201, in which the *erm*(B) gene was detected on a plasmid. Although most LAB are naturally resistant to macrolides, cases of acquired resistance among LAB have been documented, for example, in *Lactobacillus casei* strains isolated from cheese, which exhibited resistance to erythromycin [21,43]. Erythromycin resistance is generally acquired when the resistance gene is located on a plasmid [21], which aligns with our findings. The detection of such acquired resistance in LAB raises particular concern, as erythromycin is included in the “watch group” according to the WHO classification, which comprises antibiotics with a high potential for selecting resistance and of critical importance for human medicine [44]. The presence of macrolide resistance genes on mobile genetic elements in LAB strains poses a risk of horizontal transfer to other microorganisms, including clinical pathogens, representing a significant public health concern.

Phenotypic resistance to chloramphenicol and tetracyclines was detected exclusively in the *L. reuteri* KKP 4201 strain and was confirmed by the presence of the corresponding resistance genes. The *cat*(A) gene, encoding chloramphenicol acetyltransferase, was located on one plasmid, while the *tet*(W) gene, conferring resistance to tetracyclines, was found on a separate plasmid. Literature data on tetracycline resistance among LAB are variable and strain-dependent. For example, Anisimova and Yarullina [37] analyzed 34 *L. fermentum* strains isolated from human feces and dairy products—six strains exhibited phenotypic resistance to tetracycline, and all of them carried the *tet*(K) and *tet*(M) genes located on plasmids. Numerous studies have also documented a high prevalence of tetracycline resistance among LAB strains, particularly those isolated from poultry feces [35]. Other studies by Dec et al. [33,34,35] reported that phenotypic resistance to tetracyclines among LAB isolates from pigeons, turkeys, and chickens ranged from 75–84%, with *tet* genes detected in a similar proportion of isolates. Concerningly, Thumu and Halami [45] demonstrated the potential for horizontal transfer of *tet* genes from LAB to pathogenic intestinal bacteria in animals. In vivo and in vitro studies showed that LAB strains can transfer resistance traits during food fermentation, and at the end of the experiments, phenotypic tetracycline resistance was observed in *Listeria monocytogenes* and *Yersinia enterocolitica*. These findings highlight the risk of resistance dissemination in the food chain, particularly when LAB are used as food additives or probiotics.

An interesting finding in our study was the observation regarding the *lsa(*D) gene, which encodes a ribosomal protection protein. Phenotypic resistance to clindamycin was detected exclusively in the *L. reuteri* KKP 4201 strain; however, no gene conferring this resistance was identified in its genome. In contrast, the *L. petauri* KKP 4205 strain, which was phenotypically susceptible, carried the *lsa*(D) gene. Similar observations have been reported in the literature—in a study of 76 isolates (*Lactococcus formosensis, L. garvieae*, and *L. petauri*), the *lsa*(D) gene, located in the chromosomal cluster, was not found on mobile genetic elements, and phenotypic resistance was associated with an intact *lsa*(D) gene. In susceptible isolates, various mutations or insertions in *lsa*(D) were observed, likely leading to its inactivation, including internal stop codons, IS-type insertions, and substitutions in key protein motifs, such as the Walker B motif and the nucleotide-binding region. In the context of our results, it can be hypothesized that the lack of phenotypic resistance in *L. petauri* KKP 4205 strain, despite the presence of *lsa*(D), may result from similar mechanisms—i.e., the presence of mutations or structural changes in the gene that prevent proper function of the ribosomal protection protein [46].

The results obtained in our study regarding vancomycin were consistent with expectations, as susceptibility testing for this antibiotic is often not required for many LAB species due to their well-documented intrinsic resistance. Nevertheless, we performed a full phenotypic assessment, which confirmed vancomycin resistance in all analyzed strains except for *L. petauri* KKP 4205, which was susceptible to this antibiotic. The scientific literature clearly indicates that LAB exhibits a high level of natural resistance to vancomycin, although sporadic cases of susceptible strains have been reported [44]. This resistance is intrinsic and results from chromosomally encoded differences in the peptidoglycan biosynthesis pathway. The mechanism involves the replacement of the typical D-Ala–D-Ala motif in the pentapeptide of murein with D-Ala–D-lactate (conferring high-level resistance) or D-Ala–D-Ser (low-level resistance) [19]. These modifications substantially reduce the affinity of vancomycin for its target, preventing effective binding to the cell wall precursor.

In the context of our findings, the susceptibility of *L. petauri* KKP 4205 is particularly noteworthy. This strain was the only one not resistant to vancomycin, which may suggest the presence of alterations in peptidoglycan biosynthesis pathways or mutations impairing the intrinsic resistance mechanism.

## 5. Conclusions

This study provides an integrated phenotypic and genomic assessment of antimicrobial resistance in lactic acid bacteria isolated from livestock environments. By combining MIC testing with whole-genome sequencing, we demonstrated that resistance profiles in LAB cannot always be predicted from genomic data alone, underscoring the complexity of interpreting AMR in commensal bacteria. In particular, the widespread phenotypic resistance to aminoglycosides in the absence of known resistance genes highlights the importance of intrinsic mechanisms and physiological traits that may contribute to reduced antibiotic susceptibility. Conversely, the presence of ARGs without a corresponding resistance phenotype, such as the lsa(D) gene, illustrates the need for functional validation when assessing potential AMR risks in food-related microorganisms.

Importantly, our results show that LAB isolated from livestock can harbor clinically relevant ARGs, including those located on plasmids with potential for horizontal transfer. The detection of mobile elements carrying erm(B), cat(A), and tet(W) emphasizes the necessity of careful genomic screening of strains intended for use in food, feed, or probiotic applications. Such strains may serve as reservoirs of transferable resistance determinants, contributing to the broader dissemination of AMR within the agricultural ecosystem and, potentially, along the food chain.

Overall, the study highlights the value of applying both phenotypic and genomic approaches in AMR surveillance of commensal bacteria, as reliance on a single method may lead to incomplete or misleading conclusions. Continued monitoring of LAB and other members of the livestock microbiota is essential to better understand the dynamics of resistance acquisition and transfer. These findings reinforce the need for responsible antimicrobial use in animal production and support ongoing efforts to mitigate the spread of AMR at the interface of animal, environmental, and human health.

## Figures and Tables

**Table 1:** Identification of the four lactic acid bacteria strains.

Isolate Name	MALDI-TOF MS	Identification Rate (%)	*16S rRNA* Gene	Identification Rate (%)	GenBank Accession No	WGS	Closest Genome Reference	ANI (%)
KKP 4201	*L. reuteri*	99.9	*L. reuteri*	100.00	PX511705	*L. reuteri* H	GCF_020784195.1	96.29
KKP 4205	*L. garvieae*	96.7	*L. garvieae*/*petauri*	100.00	PX511764	*L. petauri*	GCF_002154895.1	98.13
KKP 4206	*Lactobacillus delbrueckii*	99.7	*L. salivarius*	100.00	PX512061	*L. oris*	GCF_001434465.1	96.10
KKP 4207	*L. * *agilis*	98.3	*L. agilis*	100.00	PX512060	*L. s agilis*	GCF_001436215.1	97.63

**Table 2:** Genome features of the four lactic acid bacteria strains.

Element	*L. reuteri* KKP 4201	*L. petauri* KKP 4205	*L. oris* KKP 4206	*L. agilis* KKP 4207
Lenght	2,306,818	2,228,894	1,993,841	2,190,702
GC content %	38.98	38.10	49.71	41.77
Genes (total)	2355	2196	2014	2182
CDSs	2230	2084	1912	2031
tRNAs	71	61	63	90
tmRNA	1	1	1	1
rRNA	18	16	15	24
ncRNAs	4	10	3	8
ncRNA Regions	28	20	20	24
ORFs	2435	2552	3320	2244
Cas arrays	0	3	0	18
Mobile elements	304	64	129	106
Plasmids	2	1	0	0

**Table 3:** Categorization of genes contained in the genomes of the four LAB strains into clusters of orthologous groups.

Clusters of Orthologous Groups	*L. reuteri* KKP 4201	*L. petauri* KKP 4205	*L. oris* KKP 4206	*L. agilis* KKP 4207
C-Energy production and conversion	63 (3.12%)	70 (3.75%)	85 (4.74%)	62 (3.49%)
D-Cell cycle control and mitosis	33 (1.63%)	27 (1.45%)	28 (1.56%)	34 (1.91%)
E-Amino acid metabolism and transport	105 (5.19%)	97 (5.19%)	98 (5.46%)	112 (6.30%)
F-Nucleotide metabolism and transport	101 (5.00%)	102 (5.46%)	97 (5.40%)	103 (5.79%)
G-Carbohydrate metabolism and transport	91 (4.50%)	118 (6.32%)	102 (5.68%)	100 (5.62%)
H-Coenzyme metabolism	59 (2.92%)	58 (3.10%)	59 (3.29%)	42 (2.36%)
I-Lipid metabolism	41 (2.03%)	34 (1.82%)	33 (1.84%)	42 (2.36%)
J-Translation	155 (7.67%)	163 (8.73%)	152 (8.47%)	160 (9.00%)
K-Transcription	133 (6.58%)	161 (8.62%)	134 (7.47%)	129 (7.26%)
L-Replication and repair	334 (16.52%)	100 (5.35%)	164 (9.14%)	164 (9.22%)
M-Cell wall/membrane/envelope biogenesis	106 (5.24%)	114 (6.10%)	109 (6.07%)	103 (5.79%)
N-Cell motility	1 (0.05%)	2 (0.11%)	1 (0.06%)	27 (1.52%)
O-Post-translational modification, protein turnover, chaperone functions	36 (1.78%)	39 (2.09%)	43 (2.40%)	39 (2.19%)
P-Inorganic ion transport and metabolism	64 (3.17%)	106 (5.67%)	63 (3.51%)	69 (3.88%)
Q-Secondary structure	9 (0.45%)	8 (0.43%)	10 (0.56%)	5 (0.28%)
T-Signal transduction	34 (1.68%)	36 (1.93%)	24 (1.34%)	35 (1.97%)
U-Intracellular trafficking and secretion	32 (1.58%)	28 (1.50%)	35 (1.95%)	32 (1.80%)
V-Defence mechanisms	31 (1.53%)	34 (1.82%)	19 (1.06%)	47 (2.64%)
S-Function unknown	354 (17.51%)	404 (21.63%)	318 (17.72%)	318 (17.89%)
R-General function prediction only	86 (4.25%)	62 (3.32%)	79 (4.40%)	78 (4.39%)
No annotation	154 (7.62%)	104 (5.57%)	142 (7.91%)	77 (4.33%)
Total (%)	2022 (100%)	1867 (100%)	1795 (100%)	1778 (100%)

**Table 4:** Minimum inhibitory concentrations (MICs) of selected antibiotics for the lactic acid bacteria strains tested.

Strains	AM	GM	KM	SM	EM	CM	TC	CL	VAN
*L. reuteri* KKP 4201 EFSA standards	>256	12	>256	>1024	>256	>256	>256	>48	>256
	2	8	64	64	1	4	32	4	n.r.
*L. petaur*i KKP 4205 EFSA standards ^1^	0.25	24	>256	>1024	0.25	2	0.5	2	1.5
	1	4	16	8	1	4	2	4	4
*L. oris* KKP 4206 EFSA standards ^2^	0.5	8	>256	48	1	0.64	16	3	>256
	4	16	64	64	1	4	8	4	n.r
*L. agilis* KKP 4207 EFSA standards ^3^	0.125	>256	>256	>1024	0.38	1.5	1	1.5	>256
	2	16	16	16	1	4	4	4	2

Note: ^1^ For other Gram-positive; ^2^ For *Lactobacillus* facultative heterofermentative; ^3^ For *Lactobacillus* obligate homofermentative; n.r.: not required; AM—ampicillin, GM—gentamycin, KM—kanamycin, SM—streptomycin, EM erythromycin, CM—clindamycin, TC—tetracycline, CL—chloramphenicol, VAN—vancomycin.

**Table 5:** Antimicrobial resistance genes detected in bacterial isolates.

Isolate Name	Gene	AMR Gene Family	Location	No. of Copies	Antibiotic/Drug Class	Mechanism	AMRFinder	CARD
*L. reuteri* KKP 4201	*van*(T) (gene in *vanG* cluster)	glycopeptide resistance gene cluster; *van*(T)	chromosome	1	glycopeptide antibiotic	antibiotic target alteration	−	+
	*erm*(B)	erm 23S ribosomal RNA methyltransferase	plasmid 1	2	macrolide antibiotic; lincosamide antibiotic; streptogramin antibiotic; streptogramin A antibiotic; streptogramin B antibiotic	antibiotic target alteration	+	+
	*cat*(A)	chloramphenicol acetyltransferase (CAT)	plasmid 1	2	phenicol antibiotic	antibiotic inactivation	+	+
	*tet*(W)	tetracycline-resistant ribosomal protection protein	plasmid 2	1	tetracycline antibiotic	antibiotic target alteration	+	+
*L. petauri* KKP 4205	*lsa*(D)	ABC-F	chromosome	1	lincosamide antibiotic; streptogramin antibiotic; streptogramin A antibiotic; pleuromutilin antibiotic	ribosome protection	+	+
	*van*(T) (gene in *vanG* cluster)	glycopeptide resistance gene cluster; *van*(T)	chromosome	1	glycopeptide antibiotic	antibiotic target alteration	−	+
	*van*(Y) *(*gene in *vanB* cluster*)*	glycopeptide resistance gene cluster; *vanB*	chromosome	1	glycopeptide antibiotic	antibiotic target alteration	−	+
	*arr*	ADP-ribosyltranseraze	chromosome	1	rifamycin	enzymatic modification	+	-
*L. oris* KKP 4206	*van*(T) (gene in *vanG* cluster)	glycopeptide resistance gene cluster; *van*(T)	chromosome	1	glycopeptide antibiotic	antibiotic target alteration	−	+
*L. agilis* KKP 4207	*van*(T) (gene in *vanG* cluster)	glycopeptide resistance gene cluster; *van*(T)	chromosome	1	glycopeptide antibiotic	antibiotic target alteration	−	+
	*qac*(G)	SMR efflux	chromosome	1	QAC(benzalkonium chloride)	efflux	−	+

**Table 6:** Phenotype and genotype of antibiotic resistance in the studied lactic acid bacteria.

Strains	Resistance Phenotype	Resistance Genotype
*L. reuteri* KKP 4201	AM, KM, GM, SM, EM, CM, TC, CL, VAN	*van*(T), *erm*(B), cat(A), *tet*(W)
*L. petauri* KKP 4205	KM, GM, SM	*lna*(D), *van*(T), *van(*Y), *arr*
*L. oris* KKP 4206	KM, TC, VAN	*van*(T)
*L. agilis* KKP 4207	KM, GM, SM, VAN	*van*(T), *qac(*G)

## Data Availability

The datasets generated during the current study are available in the GeneBank NCBI repository, Bioproject ID: PRJNA1362993 (http://www.ncbi.nlm.nih.gov/bioproject/1362993, accessed on 6 November 2025). Further inquiries can be directed to the corresponding authors.

## Abbreviations

The following abbreviations are used in this manuscript:

α-CHCA	α-cyano-4-hydroxycinnamic acid
AGPs	antibiotic growth promoters
AMR	antimicrobial resistance
ANI	Average nucleotide identity
ARGs	antibiotic resistance genes
FCR	feed conversion ratio
HGT	horizontal gene transfer
KKP	Collection of Industrial Microorganisms—Microbiological Resources Center
LAB	lactic acid bacteria
MDR	multidrug resistant
MIC	minimum inhibitory concentration
PBS	phosphate-buffered saline
WHO	World Health Organization
WGS	whole-genome sequencing
